# Comparative AI-optimized HPLC–DAD strategy for the simultaneous determination of ranolazine, amlodipine, and diltiazem with pharmacotherapeutic relevance and multi-trait sustainability assessment

**DOI:** 10.1038/s41598-026-48679-w

**Published:** 2026-04-25

**Authors:** Sara I. Aboras, Mohamed A. Korany, Randa A. Yehia, Marwa A. A. Ragab

**Affiliations:** Department of Pharmaceutical Analytical Chemistry, Faculty of Pharmacy, Alexandria University, Alexandria, Egypt

**Keywords:** Ranolazine, Amlodipine, Diltiazem, HPLC–DAD, Artificial intelligence, Multi-color assessment tool, Chemistry, Drug discovery

## Abstract

A robust and reliable HPLC–DAD method was developed and validated for the simultaneous determination of ranolazine, diltiazem, and amlodipine, a drug combination relevant to the concurrent management of chronic angina and hypertension. Preliminary screening of chromatographic conditions was supported by an AI-assisted platform and subsequently refined through conventional experimental optimization. The proposed method was successfully applied to bulk materials and combined dosage forms. It was further extended to spiked rat plasma to verify its applicability for monitoring ranolazine in the presence of co-administered diltiazem and amlodipine. Chromatographic separation was achieved on a C18 column using an isocratic mobile phase consisting of buffer (pH 5), acetonitrile, and methanol (60:30:10, v/v) at a flow rate of 1.0 mL/min. The developed method provided efficient separation with satisfactory peak symmetry and resolution under optimized conditions. The AI-assisted predictions showed good agreement with experimentally optimized parameters, supporting their utility as a preliminary tool to guide method development. The method was validated in accordance with established guidelines, demonstrating satisfactory linearity, accuracy, precision, and selectivity within the studied concentration range. The analytical performance and environmental impact were evaluated and compared with previous methods using the Multi-Color Assessment tool, yielding an overall Whiteness Score of 64.8% overwhelming the other reported methods. In addition, the alignment of the AI-optimized method with sustainability principles was assessed using the Need, Quality, and Sustainability tool and compared with conventional optimization. The results demonstrate that the proposed method provides a reliable and environmentally considerate analytical approach for the simultaneous quantification of the studied drug combination in pharmaceutical formulations with demonstrated applicability in biological matrices.

**Supplementary Information:**

The online version contains supplementary material available at 10.1038/s41598-026-48679-w.

## Introduction

**Fig. 1:**
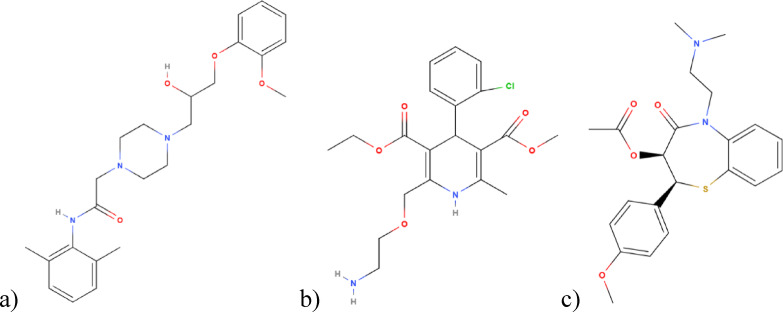
Chemical structures of (**a**) ranolazine (RNZ), (**b**) amlodipine (AMD), and (**c**) diltiazem (DTZ).

With 19.8 million deaths in 2022, cardiovascular diseases (CVDs) represent a significant global health burden and consequently they become the leading cause of death worldwide^1^. Among these, chronic angina and hypertension are highly prevalent, often coexisting indeed, and typically necessitate long-term pharmacological management through multidrug regimens targeting different physiological mechanisms^2^. Ranolazine (RNZ) with amlodipine (AMD), or diltiazem (DTZ), Fig. 1, are frequently co-administered in clinical settings due to their complementary therapeutic profiles in severe chronic angina patients who suffered from hypertension^3–5^. RNZ administered twice daily has been shown to improve exercise tolerance and provide additional antianginal and anti-ischemic benefits in patients with severe chronic angina who remain symptomatic despite receiving standard therapy with AMD or DTZ. Importantly, these benefits occur with minimal hemodynamic effects and without evidence of adverse long-term survival outcomes over 1–2 years of treatment. Consequently, the co-administration of RNZ and AMD or DTZ is particularly beneficial in patients with chronic stable angina, hypertension with coexisting arrhythmias, or those who do not adequately respond to monotherapy^3–5^.

RNZ, a selective inhibitor of the late inward sodium current, improves myocardial relaxation and oxygen efficiency without significantly affecting heart rate or blood pressure in chronic angina patients^5,6^. AMD, a long-acting dihydropyridine calcium channel blocker, induces vasodilation and reduces peripheral resistance^7^, while DTZ, a benzothiazepine calcium channel blocker, provides both vasodilatory and negative chronotropic effects^8^.

Given the clinical significance of this combination, developing an efficient analytical method capable of simultaneously quantifying RNZ, AMD, and DTZ is essential for pharmaceutical quality control and pharmacokinetic studies. However, the simultaneous analysis of these three drugs poses several analytical challenges due to their diverse physicochemical properties and overlapping spectral features.

To date, various methods have been reported for the individual analysis of these drugs or along with other drugs. However, reports focusing on the simultaneous analysis of all three drugs as ternary mixtures are absent except for a few studies analyzing only AMD with DTZ^9^. Nevertheless, RNZ, AMD, and DTZ were analyzed either separately or with other drugs using HPLC, UV, CE or HPTLC.

RNZ was determined by few analytical methods: HPLC method, along with its enantiomer, in tablets^10^ and LC-MS–MS in human plasma^11^. Also, RNZ was analyzed by UV- Spectrophotometry in extended-release tablets^12^.

AMD has been detected by various analytical techniques: HPLC-DAD method in formulations^13^, HPTLC with other drugs^14^ and CE-DAD with Atorvastatin^15^. Also, it is determined by spectrophotometry in dosage form^16^ and spectrofluorimetry in different matrices^17,18^.

DTZ is determined by multiple analytical methods: HPLC and HPTLC-DAD methods along with lidocaine in dosage form^19,20^, capillary electrophoresis with other drugs in human serum^21^ and LC-MS/MS method beside its metabolite in human plasma^22^. Also, it is determined by spectrophotometry^23^ and spectrofluorimetry^24^.

The quantification of multi-component drug combinations in pharmaceutical formulations or biological matrices requires carefully optimized chromatographic methods. Recently, artificial intelligence (AI)–assisted platforms such as HPLC-Method-Developer have been explored as supportive tools for screening chromatographic parameters, including column chemistry, mobile phase composition, and detection conditions, thereby facilitating the initial stages of method development^25,26^.

In analytical chemistry, it is important to distinguish greenness from sustainability. Greenness focuses on reducing hazardous reagents, solvent consumption, and waste generation, whereas sustainability considers broader aspects such as analytical performance, robustness, practicality, and resource efficiency. In this work, these aspects were evaluated within the framework of White Analytical Chemistry using the Multi-Color Assessment (MA) tool, which integrates several established metrics to provide a comprehensive sustainability evaluation. Additionally, the alignment of the developed method with the United Nations Sustainable Development Goals (SDGs) was assessed using the Need, Quality, and Sustainability (NQS) tool^27–31^.

In light of these considerations, this work presents a validated HPLC–DAD method capable of simultaneously resolving and quantifying RNZ, AMD, and DTZ in bulk, pharmaceutical dosage forms, and spiked rat plasma. The method employs isocratic elution with minimal sample preparation and was developed in accordance with ICH guidelines, providing a reliable approach for routine pharmaceutical analysis. To the best of our knowledge, no previous study has reported the simultaneous determination of these three drugs in a single analytical run which is also combined with a structured sustainability assessment. Accordingly, the proposed method integrates chromatographic optimization with multi-dimensional sustainability evaluation within a White Analytical Chemistry framework, incorporating AI-assisted method development to support efficient and sustainable analytical design.

## Experimental

###  Instrumentation

The HPLC-DAD analyses were conducted on an Agilent 1260 series system (Agilent Technologies, Santa Clara, CA, USA), comprising a quaternary pump (G1311C), autosampler (G1329B), column thermostat (G1316A), degasser (G1322A), and diode array detector (G1315C) operated via ChemStation software (C.01.07). Separation utilized a Zorbax Eclipse Plus C18 column (4.6 × 100 mm, 3.5 μm). In the plasma assay, the organic phase was concentrated to dryness under vacuum using a Christ rotational vacuum concentrator (Germany).

### Materials and reagents

RNZ was purchased from Sigma-Aldrich^®^ whereas DTZ and AMD besylate were supplied from Amriya Pharmaceutical Industries, Alexandria, Egypt. The commercially manufactured dosage forms were bought from the Egyptian market as follows: RANEXA ^®^ from Menarini Group, containing 375 mg RNZ, DELAY TIAZIM SR^®^ from GlaxoSmithKline, containing 90 mg DTZ and NORVASK^®^ tablets from Pfizer company, containing 10 mg AMD besylate. The HPLC-grade methanol (MeOH) and HPLC-grade acetonitrile (ACN) were bought from Baker’s in Ireland. Phosphate buffer (sodium dihydrogen phosphate monohydrate), NaOH, and phosphoric acid were bought from El-Nasr Chemical Industry Company in Egypt.

### Preparation of stock standard solutions

Stock solutions of RNZ, DTZ and AMD, at concentration 1000 µg/mL for the three dugs, were prepared in HPLC-grade methanol. These solutions were stored at 4 °C in the refrigerator for one week.

### Construction of calibration curves

To prepare a calibration range of 1–40 µg/mL for RNZ, DTZ, and AMD, appropriate aliquots of each stock solution were accurately transferred into 10-mL volumetric flasks and diluted to volume with the mobile phase mixture.

### Synthetic mixtures

Precise amounts of RNZ, DTZ and AMD stock solutions were transferred to a set of 10 mL-volumetric flasks. Then, the volumetric flasks were filled to the mark with mobile phase ratio to prepare three synthetic mixtures of the corresponding concentrations: 5, 15 and 30 µg/mL, 30, 5 and 15 µg/mL and 15, 30 and 5 µg/mL were prepared for RNZ, DTZ and AMD, respectively.

### Preparation of dosage form samples

An accurately weighed portions of the finely powdered dosage forms (RANEXA ^®^, DELAY TIAZIM SR^®^ and NORVASK^®^) equivalent to 40 mg RNZ, 9 mg DTZ, and 1 mg AMD transferred into a 100 mL volumetric flask, respectively. About 70 mL of methanol was added, and the mixture was sonicated for 15 min to ensure complete dissolution of the active ingredients. The volume was then adjusted to the mark with methanol and thoroughly mixed. The resulting solution was filtered through a 0.45 μm PTFE membrane filter to remove any insoluble excipients. Appropriate aliquots of this filtrate were further diluted with the mobile phase to obtain concentrations within the linear ranges of the proposed method corresponds to 40, 9, 1 µg/mL RNZ, DTZ and AMD, respectively. The prepared solution was analyzed in five replicates using the optimized chromatographic conditions. The concentrations of RNZ, DTZ and AMD were determined using their corresponding regression equations, and the results were expressed as a percentage of the labeled claim for each drug.

### Chromatographic conditions

The mobile phase isocratic system used is 20 mM sodium phosphate buffer of pH 5 (sodium dihydrogen phosphate monohydrate), ACN and MeOH in a ratio 60:30:10 (v/v). The 20 mM phosphate buffer was prepared by dissolving sodium dihydrogen phosphate monohydrate in distilled water, followed by adjustment of the pH to 5.0 using dilute phosphoric acid or sodium hydroxide. The prepared buffer was filtered through a 0.45 μm membrane filter and degassed prior to use in the mobile phase. The flow rate is 1.0 mL/minute and the injection volume of 20-µL at 25 °C. The mobile phase passes through 0.45 μm Millipore filter in order to be filtered prior to use. The photodiode array detector was set at the characteristic wavelengths of each drug — 270 nm for RNZ and 240 nm for both AMD and DTZ— to ensure maximum sensitivity and selectivity.

### Animals

Adult male Wistar rats (200–250 g), aged 8–10 weeks, were used in this study. The animals were obtained from the animal house facility of the Faculty of Pharmacy, Alexandria University, Alexandria, Egypt. The animal study was conducted in accordance with the ARRIVE guidelines and relevant institutional and international regulations, and was approved by the Institutional Animal Care and Use Committee (IACUC) of Alexandria University, Egypt with number (AU 0620261171303).

For blood sampling, rats were anesthetized using inhalational isoflurane (3–4% for induction and 1–2% for maintenance in oxygen). Approximately 2 mL of blood was collected from the retro-orbital venous plexus using unheparinized glass capillary tubes and transferred into EDTA-precoated tubes. Samples were centrifuged at 7000 rpm for 15 min, and the plasma supernatant was separated and stored at − 80 °C until analysis.

At the end of the experimental procedures, animals were humanely euthanized by an overdose of isoflurane (≥ 5%), ensuring rapid loss of consciousness and death, in accordance with IACUC recommendations.

### Procedures for spiked rat plasma

For plasma application, a mixed working standard solution of RNZ, DTZ and AMD was prepared at concentrations 100 µg/mL for all drugs. A 100 µL sample of drug-free rat plasma was spiked with 50 µL of the mixed working standard solution of RNZ, DTZ and AMD, in a 1.5 mL Eppendorf tube. To this mixture, 300 µL of methanol was added as a protein-precipitating and extracting solvent, followed by 2-minutes vortex. The resulting mixture was centrifuged at 7000 rpm (≈ 11,200×g) for 10 min to ensure complete separation of the precipitated proteins. The obtained clear supernatant, representing plasma spiked with the studied drugs, was transferred to a tube then evaporated to dryness. The residue was then reconstituted in 400 µL mobile phase followed by sonication and filtration using 13 mm PTFE of pore size 0.2 μm to obtain final concentration of 12.5 µg/mL for the studied drugs. The filtrate was finally subjected to the proposed HPLC-DAD procedure. With the same manner, 1 and 5 µg/mL were prepared to ensure the method sensitivity. A blank plasma sample, treated in the same manner but without drugs’ addition, was also processed to obtain the baseline chromatogram. For testing extraction efficiency, a standard solution was prepared following the same procedure but without plasma.

## Results and discussion

The proposed HPLC method aimed to develop a reliable chromatographic system capable of concurrently separating and eluting RNZ, DTZ and AMD in reasonable retention times with optimal peaks shape and resolution and avoiding interference from endogenous plasma peaks. In our study, the analytical method was first optimized using traditional trial-and-error approaches to establish baseline chromatographic performance. This conventional optimization provided a reference framework for evaluating the efficiency of the AI-assisted HPLC method development platform. Subsequently, the same experimental design was introduced into the HPLC-Method-Developer tool, which automatically suggested optimized chromatographic parameters based on multi-variable computational modeling. Remarkably, the optimized conditions predicted by the AI tool yielded comparable, and in some aspects superior, separation performance to the conventionally derived method. This demonstrates the powerful potential of artificial intelligence in rationalizing chromatographic optimization, reducing experimental workload, and enhancing method robustness.

### Conventional development and optimization of the chromatographic method

The following experimental parameters were tested, in the traditional way, to maximize the sensitivity and selectivity of the analytical procedure:

#### Stationary phase

Two reversed-phase columns were evaluated: C8 (4.6 × 220 mm, 5 μm) and C18 (4.6 × 100 mm, 3.5 μm). The C18 column achieved baseline separation of RNZ, DTZ, and AMD within a short run time, whereas the long C8 column produced late elution (≈ 15 min) with tailing peaks, consistent with performance deterioration of an aged column, Fig. S1.

#### Mobile phase

The chromatographic separation of the studied analytes was interpreted based on their physicochemical properties, particularly lipophilicity and ionization characteristics. In reversed-phase liquid chromatography, retention is primarily governed by hydrophobic interactions between analytes and the non-polar stationary phase. The investigated drugs exhibit low to moderate lipophilicity, with reported log P values of approximately 2.07 for RNZ, around 2.8 for DTZ, and about 3.04 for AMD^32^. Accordingly, the more lipophilic compound tends to interact more strongly with the stationary phase, resulting in longer retention.

The peaks shape of RNZ, DTZ, and AMD were significantly influenced by the type of mobile phase. Two organic solvents, ACN and MeOH had been explored. Different ratios of MeOH were tried in isocratic elution with buffer at pH 5. Starting with 50% MeOH, RNZ and DTZ were eluted in proper retention time and peak shape. However, AMD were eluted very late with a very tailed broadened peak. Therefore, shifting to ACN instead of MeOH was tried to enhance AMD elution and it was successful but resulted in overlapping of RNZ and DTZ. The combination of ACN and MeOH as organic modifiers allowed fine tuning of the elution strength and selectivity, enabling efficient resolution of RNZ, DTZ, and AMD within a short analysis time. Consequently, trying different ratios of MeOH and ACN along with buffer was essential. After a number of trials, using buffer: MeOH: ACN in a ratio 60:10:30 gave the best elution regarding elution time and peak shape of RNZ, DTZ and AMD at 2.79, 3.72 and 5.15 min with tailing factor 1.15, 1.89 and 1.89, respectively. The total run time was 6 min.

#### pH of the aqueous phase

Chromatographic selectivity is influenced by the ionization state of the analytes. Thus, aqueous portion of the mobile phase was optimized regarding different factors; pKa of the drugs, retention times and peaks shape. The pKa values of the studied drugs were 7.2, 7.5, and 8.7 for RNZ, DTZ and AMD, respectively^32^. Therefore, adjusting the mobile phase to pH 5 using phosphate buffer helps control the protonation state of these weakly basic compounds. Under these conditions, trying acidic pH values was crucial to avoid late elution and peak splitting in neutral and alkaline pH values (7–9) and thus improving peak symmetry and reproducibility in reasonable run time. We tried pH 5 and 3 and both gave comparable results. Thus, pH 5 was chosen due to its milder acidity for better column lifetime and instrument protection from corrosion.

#### Detection wavelength

**Fig. 2:**
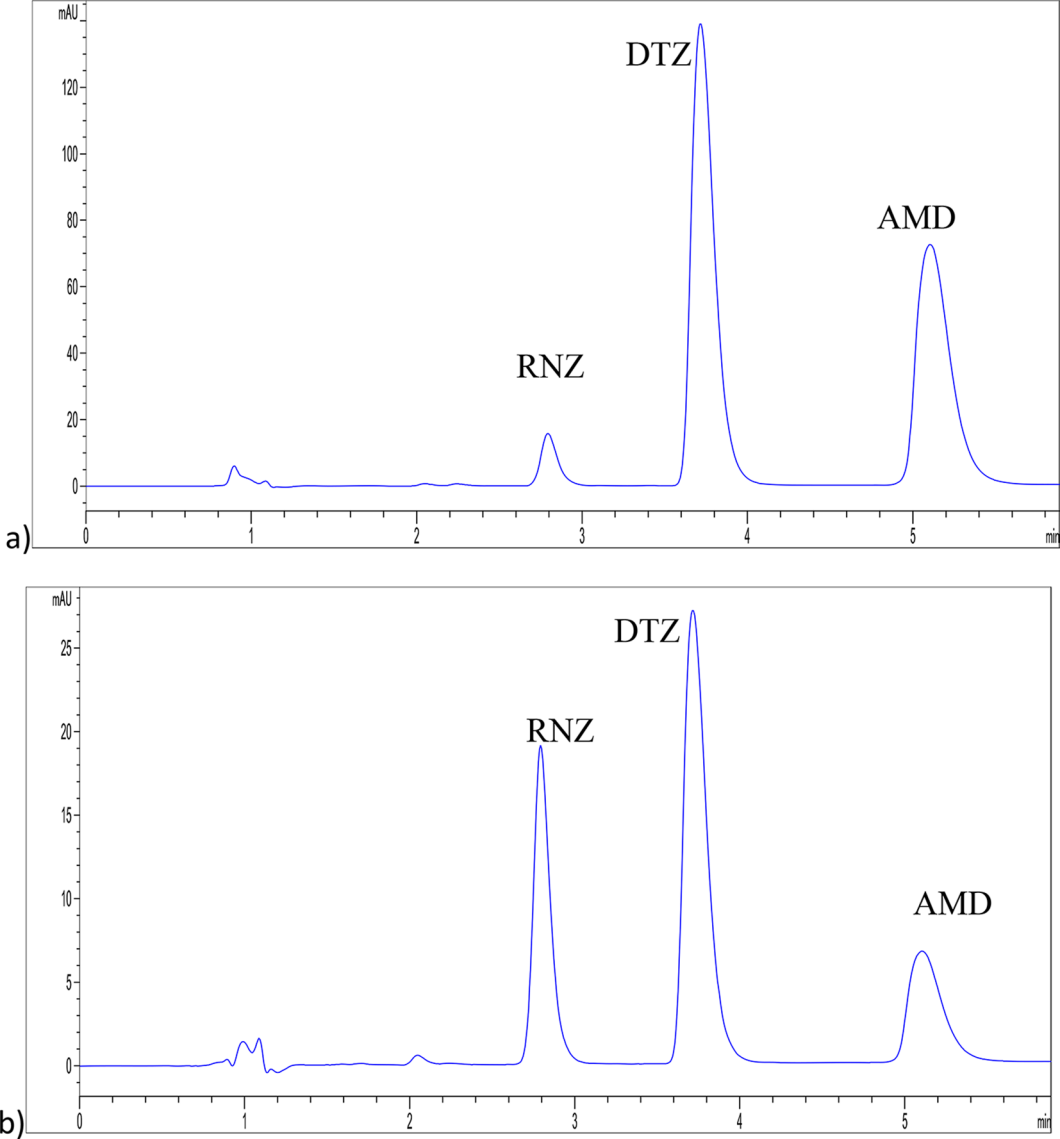
HPLC chromatograms of 20 µg/mL of RNZ, DTZ, and AMD at (**a**) 240 nm for AMD and DTZ quantitation and (**b**) 270 nm for RNZ determination.

In order to increase the sensitivity of the approach, various wavelengths were explored for the measurement of the recommended medications. The detection at 270 nm for RNZ, 240 nm for both DTZ, and AMD yielded the highest sensitivity as they are the λ _max_ for each one, Fig. 2.

The significant separation of RNZ, DTZ, and AMD is shown in Fig. 2, which also shows peak sharpness and resolution within an acceptable runtime. Table 1 shows that the method’s chromatographic separation system suitability parameters satisfy USP regulations^33^.

**Table 1:** System suitability parameters for the HPLC-DAD determination of RNZ, DTZ and AMD.

	RNZ (270 nm)	DTZ (240 nm)		AMD (240 nm)
t _*R*_ ± SD (min)^b^	2.79 min ± 0.01	3.72 min ± 0.01		5.15 min ± 0.01
Plates (N)	2274	2082		2547
Resolution ^c^	3.73		4.40	
Capacity factor (k’)	2.1	3.1		4.8
Tailing Factor	1.15	1.89		1.89
Selectivity factor (α)	1.5		1.5	

^a^ System suitability recommendations: k’ (2–10), *N* > 2000, α > 1, R_s_ > 2 and T_f_ ≤2^33^.

^b^ Average t_R_ ± SD of three determinations.

^c^ Resolution between RNZ, DTZ and AMD.

### AI-Based development and optimization of the chromatographic method

In parallel to traditional way of method optimization through trial and error, AI HPLC Method Developer tool, powered by ChatGPT-4o^25^, was validated to propose chromatographic conditions suitable for elution the studied drugs. This specialized GPT-based model is presumed to have been trained on extensive chromatographic method datasets and analyte physicochemical profiles. For this work, input data included the chemical names of RNZ, AMD, and DTZ, with the best available column, C18 column (100 mm × 4.6 mm, 3.5 μm), for sustainability goals to achieve simultaneous separation for drugs analysis in shortest run time. As we specified that we wished isocratic elution system, the AI tool proposed the following starting conditions on the specified column: isocratic elution 35% acetonitrile with 65% phosphate buffer 20 mM (pH 3.0), a flow rate of 1.0 mL/min, detection at 235 nm, and a run time of 15 min. Moreover, this tool suggests, if resolution or peak shapes are inadequate; adjusting organic strength in small steps (± 2–3% ACN), fine‑tuning pH, switching organic modifier to MeOH or use ACN: MeOH blends (e.g., 25:10 ACN: MeOH at same total % organic). Clearly, we can find that both optimization methods gave similar chromatographic conditions. However, the system, proposed by AI, required 2 or 3 modification steps to reach the optimum conditions, focusing on resolution, peak symmetry, and analysis time, (≈ 2 h, 50mL organic solvents) unlike the traditional way that consumed larger numbers of trials (≈ 2 days, 500mL organic solvents). This proves that this AI tool can help saving time and effort beside waste and thus make analytical methods more sustainable^25^. 

However, as the tool’s training data, algorithms, and internal weighting remain proprietary and undisclosed, the approach represents a “black box” model. Consequently, experimental verification was required to evaluate the real-world feasibility and analytical performance of the predicted method.

### Methods validation

Validation of the proposed methods was carried out in accordance with ICH guidelines^34^ as detailed below:

#### Linearity

The selected calibration range (1–40 µg/mL) was chosen to adequately cover the concentration levels expected during pharmaceutical dosage form analysis after appropriate dilution. The plasma application was intended to demonstrate matrix compatibility and selectivity rather than trace-level pharmacokinetic quantification.

Six data points and triplicate injections of standards at each concentration level within the linearity range were used to create a regression line for RNZ, DTZ, and AMD, Table 2. Plotting of peak areas against RNZ, DTZ, and AMD concentrations was done. The findings demonstrated a high degree of linearity (*r* = 0.9990, 0.9991, 0.9992) between the peak regions of RNZ, DTZ and AMD and the corresponding concentrations, respectively. High (F) values for RNZ, DTZ, and AMD were 1445.04, 1583.02, and 1787.18, respectively, indicating good linearity^35^. 

**Table 2:** Analytical parameters for determination of RNZ, DTZ and AMD using the proposed HPLC method.

Analyte	RNZ	DTZ	AMD
Wavelength (nm)	270	240	240
Linearity Range (µg/mL)	1–40		
LOD (µg/mL)	0.24	0.22	0.28
LOQ (µg/mL)	0.80	0.76	0.96
Correlation coefficient	0.9990	0.9991	0.9992
S_y/x_^a^	19.32	17.36	14.17
Slope	22.31	20.99	18.20
SE of slope	0.58	0.52	0.43
Intercept	49.06	14.73	10.66
F ^b^	1445.04	1583.02	1787.18
Significance F ^c^	4.00 × 10^− 5^	3.49 × 10^− 5^	2.91 × 10^− 5^
Accuracy (% recovery)^d^	100.12	99.84	100.05
Repeatability precision (RSD)^e^	0.48	0.39	0.31
Intermediate precision (RSD)^e^	0.71	0.65	0.54

^a^ Average of 9 determinations (3 concentrations repeated 3 times).

^b^ RSD of 9 determinations (3 concentrations repeated 3 times).

^c^ The standard deviation of the residuals.

^d^ F-statistic obtained from regression analysis.

^e^ corresponding p-value assessing statistical significance of regression.

#### Detection and quantitation limits

The practical determination of the limit of detection (LOD) and limit of quantitation (LOQ) involved estimating the analyte concentration with a signal-to-noise ratio of 3:1 for LOD and 10:1 for LOQ. The low LOD and LOQ attest to the suggested HPLC method’s sufficient sensitivity. Table 2 shows that for RNZ, DTZ, and AMD, the LOD was 0.24, 0.22, and 0.28, while the LOQ was 0.80, 0.76, and 0.96 µg/mL, respectively.

#### Accuracy

Analyzing synthetic mixes of RNZ, DTZ, and AMD at different concentration ratios within each drug’s working range allowed for the evaluation of the suggested method’s accuracy. The prepared concentrations were 5, 15 and 30 µg/mL, 30, 5 and 15 µg/mL and 15, 30 and 5 µg/mL were prepared for RNZ, DTZ and AMD, respectively. The mean of the concentrations and the percentage relative error for RNZ, DTZ, and AMD were used to express accuracy. The accuracy of the approach for the synthetic mixes is shown in Table 2. The method’s great accuracy was demonstrated by the small relative errors, Table 2.

#### Precision

The prepared synthetic mixtures comprising the three medications at varying concentration levels were used to determine the intra-day and inter-day variance for RNZ, DTZ, and AMD. RSD percentage values obtained by performing the assay three times on the same day for intra-day precision were used to determine the method’s repeatability. The assay of the sample sets on three separate days (inter-day precision) was used to evaluate intermediate precision. The suggested strategy offered acceptable intra-day and inter-day variance of RNZ, DTZ, and AMD, according to the RSD percentage values for all mixture ratios shown in Table 2 (less than 2%).

#### Selectivity

**Fig. 3:**
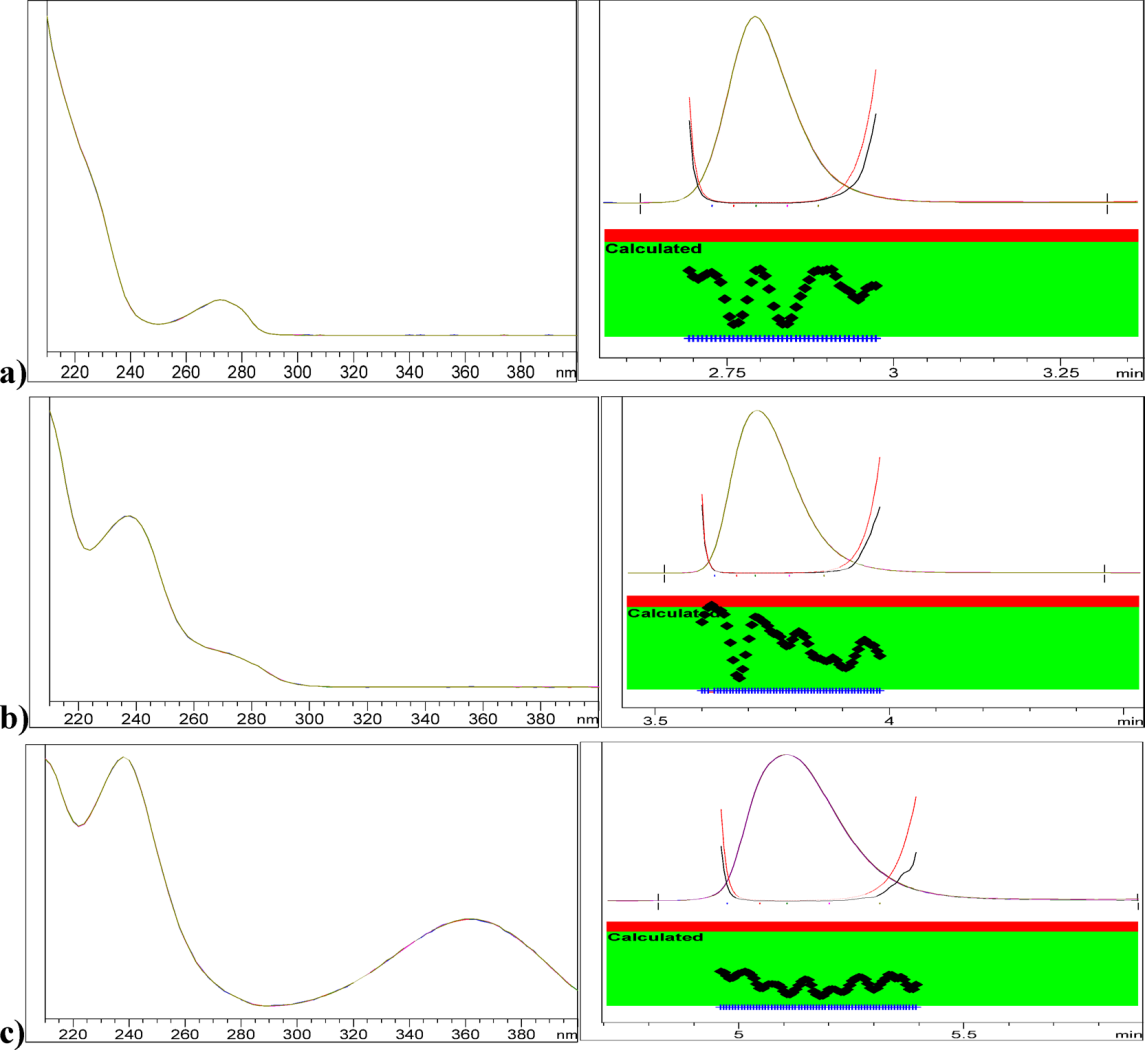
Purity profiles and plots of RNZ (**a**), DTZ (**b**) and AMD (**c**).

Selectivity was evaluated in RNZ, DTZ and AMD to ensure absence of interferences. Consistently high mean recoveries (100% ± 2%) were obtained for RNZ, DTZ, and AMD using the chromatographic method, with RSD values maintained within ± 2% across various proportions. Moreover, HPLC–DAD analysis verified peak purity, confirming the specificity for RNZ, DTZ, and AMD, as shown in Fig. 3.

#### Stability of solutions

The stability of RNZ, DTZ, and AMD stock solutions was evaluated after storage at 4 °C for one week. Stability was confirmed by the absence of significant changes in chromatographic peak area, retention time, or peak purity profiles of the analytes. Furthermore, no additional interfering peaks were detected in the chromatograms, indicating that the stock solutions remained stable under the tested storage conditions.

#### Robustness of the HPLC method

Method robustness was examined by re-measuring RNZ, DTZ, and AMD at the previously defined concentrations, applying small intentional changes to temperature, wavelength, and pH. The results are summarized in table S1. It was found that the assay of RNZ, DTZ and AMD was not significantly affected by small deliberate changes to the parameters. Retention times (SD ≤ 2%) and/or essentially unchanged responses (peak areas 100 ± 2%) for RNZ, DTZ and AMD in the HPLC technique demonstrate satisfactory resilience.

#### Analysis of dosage forms

The obtained assay results demonstrated excellent agreement with the nominal labeled claims of the tested dosage form, confirming the accuracy and reliability of the proposed HPLC-DAD method for routine quality control analysis. The recoveries for RNZ, DTZ and AMD were found to be within the acceptable pharmaceutical limits 98.56, 101.25, and 99.32%, respectively, indicating negligible interference from excipients commonly present in the formulation matrix, Table S2. The well-resolved chromatographic peaks, absence of additional interfering signals, and consistent retention times further validated the method’s selectivity and robustness, Fig. S2. Moreover, the low %RSD values, 0.89, 0.55, and 1.63 for RNZ, DTZ and AMD, respectively, across replicate analyses confirmed the precision of the method providing a reliable tool for quality control laboratories without the need for tedious sample pretreatment or expensive instrumentation.

### Application to spiked plasma

Co-administration of RNZ with calcium channel blockers such as DTZ or AMD is beneficial in the management of chronic stable angina, hypertension with coexisting arrhythmias, or in patients inadequately controlled on monotherapy^3–5^. To extend the applicability of the developed HPLC–DAD method, it was applied to the simultaneous determination of RNZ, DTZ, and AMD in spiked rat plasma. The plasma experiment was designed to demonstrate matrix compatibility and chromatographic selectivity rather than to establish a fully validated bioanalytical method. Accordingly, a simple protein precipitation procedure was adopted as a preliminary sample preparation step without the incorporation of an internal standard.

**Fig. 4:**
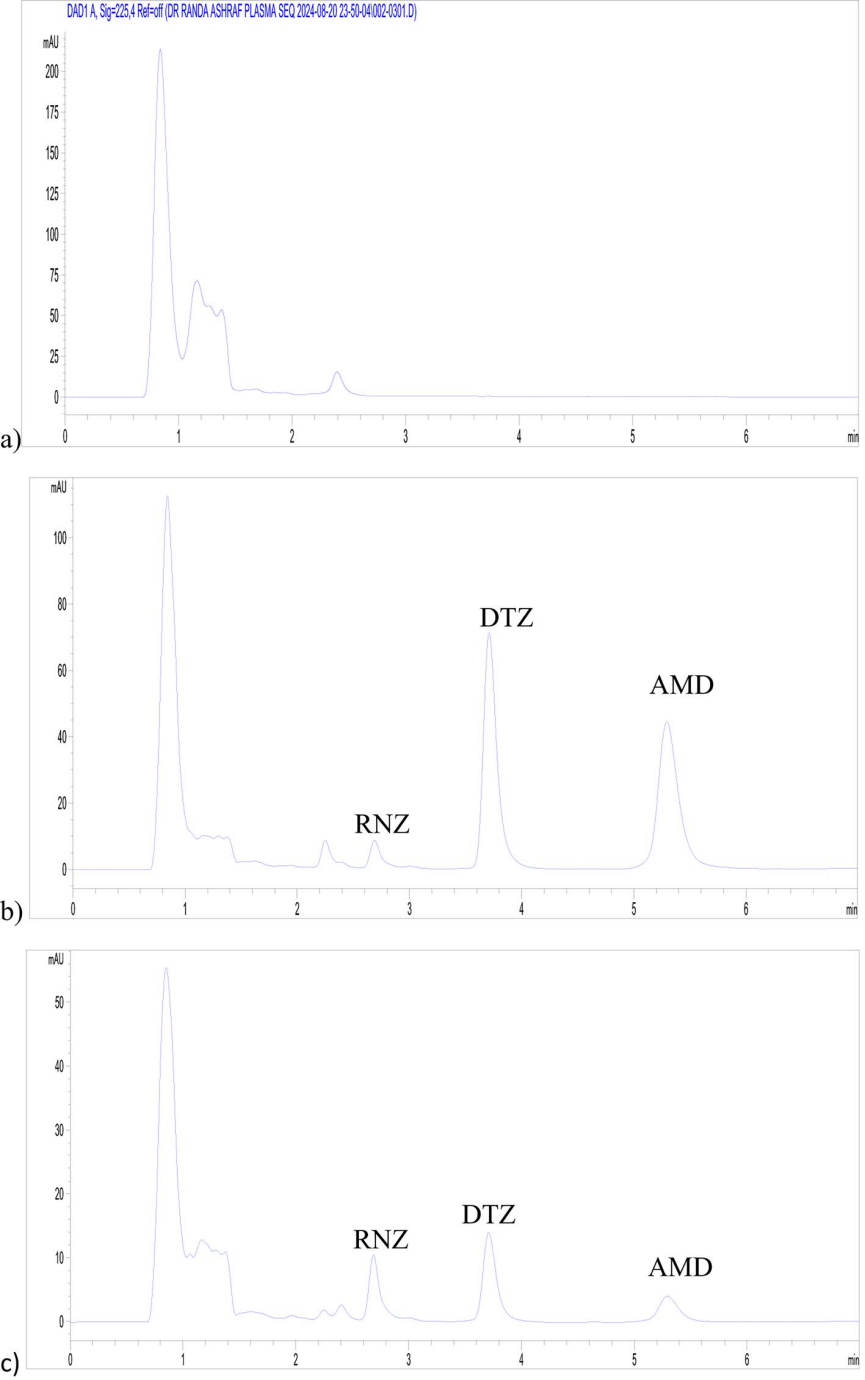
Chromatograms showing (**a**) blank plasma and spiked plasma with the studied drugs at 12.5 µg/mL measured at (**b**) 240 nm and (**c**) 270 nm.

The extraction conditions were optimized by evaluating methanol and acetonitrile as protein precipitating agents. Methanol provided superior performance in terms of peak shape, extraction efficiency, and tailing factor, and was therefore selected for subsequent analysis (Fig. 4 and Fig. S3). The extraction efficiency, expressed as percentage recovery ± RSD%, was 96.51 ± 0.95% for RNZ, 98.41 ± 1.12% for DTZ, and 85.58 ± 1.48% for AMD, confirming the reliability of the proposed method for their determination in rat plasma. The peak purity plots further confirmed the absence of endogenous plasma interferences, demonstrating high selectivity and specificity (Fig. S4).

To further evaluate method sensitivity, chromatograms of spiked plasma at lower concentrations (1 and 5 µg/mL) were recorded (Figs. S5 and S6), demonstrating the applicability of the method for monitoring RNZ levels at concentrations below its reported peak plasma concentration (Cmax). Reported Cmax values following oral administration are approximately 2–6 µg/mL for RNZ, 100–300 ng/mL for DTZ, and 5–15 ng/mL for AMD, depending on dose and formulation^6,36,37^. Notably, the developed method exhibited sufficient sensitivity for the determination of RNZ within its clinically relevant concentration range in the presence of DTZ and AMD. However, for DTZ and AMD, whose plasma levels are considerably lower, further sensitivity enhancement would be required for full pharmacokinetic applications. Therefore, the present study emphasizes matrix compatibility and simultaneous multi-analyte determination rather than ultra-trace bioanalytical quantification.

### The multi-color assessment (MA) tool

In alignment with the principles of White Analytical Chemistry (WAC), the Multi-Color Assessment (MA) Tool was employed as an integrated web-based platform for comprehensive method evaluation. This tool unifies four established frameworks—the Green Evaluation Metric for Analytical Methods (GEMAM), the Blueness Assessment Graphical Index (BAGI), the Redness Analytical Performance Index (RAPI), and the Violet Innovation Grade Index (VIGI)—within a structured 51-item protocol that simultaneously assesses environmental impact, analytical performance, operational feasibility, and innovation. By linking sustainability assessment with Analytical Quality by Design (AQbD) concepts, the platform provides instantaneous scoring and graphical outputs, four-segment typographic “M”, ultimately generating an overall “Whiteness Score”, white “A,” that reflects the comprehensive sustainability of the method. In this study, the developed HPLC–DAD method achieved a whiteness score of 64.8%, derived from individual scores of 66.7 (GEMAM), 72.5 (BAGI), 70 (RAPI), and 50 (VIGI), indicating balanced analytical performance and sustainability using AI tool, with high throughput (10 analyses per hour) together with its successful biological application, contributing to improved RAPI and BAGI scores and thus efficiency and practical applicability, respectively, Table 3^29^. 

**Table 3:** Comparison between MA Tool for our proposed method, RNZ reported method and DTZ & AMD reported method.

	Proposed method	Reported method for RNZ	Reported method for DTZ & AMD
Letter M			
Letter A			

To highlight the sustainability advantages of the proposed method, its performance was systematically compared with previously reported analytical approaches. The proposed method demonstrated superior WAC performance, achieving a Whiteness Score of 64.8%, compared to previously reported methods such as LC–MS/MS approach for RNZ in plasma^11^ (52.8%) and multi-component analysis for DTZ and AMD^9^ (52.8%). This enhanced performance can be attributed to improved greenness characteristics, including reduced solvent consumption, lower energy requirements, and simplified analytical procedures, in addition to the incorporation of supportive AI-assisted tools during method development which enhanced VIGI score. In contrast, the compared methods typically involve more complex instrumentation, higher solvent usage, and greater energy demand, which negatively impact their overall sustainability profiles. Accordingly, the proposed method offers a more efficient and environmentally considerate analytical alternative, aligning with the principles of WAC through a balanced integration of analytical performance, sustainability, and innovation.

### Comparative sustainability assessment of conventional and AI optimized methods using the NQS index

This study presents both a conventional HPLC method and an AI-optimized counterpart for multi-drug analysis, demonstrating their contribution to global sustainability objectives^30,31^. As shown in Table 4, the proposed approaches were assessed against selected United Nations Sustainable Development Goals (SDGs), highlighting their role in promoting more sustainable practices within analytical sciences. The AI-optimized method achieved a sustainability score of 53%, fulfilling 9 out of the 17 SDGs, thereby indicating a notable advancement in sustainable analytical methodology. In comparison, the conventional HPLC approach satisfied only 5 SDGs and did not meet others, particularly SDGs 8, 11, 12 and 13, owing to its reliance on larger volumes of acetonitrile during repeated optimization trials, which increased both cost and solvent waste beside time loss. Consequently, the conventional method attained a lower sustainability score of 29%.

The analytical need of the developed methods was evaluated according to Koel’s pyramid^38^, which classifies analytical techniques based on their usefulness and resource requirements. At the base of the pyramid are simple, widely accessible, and energy-efficient tools with a demand level of 100%, followed by automated, high-throughput systems at 75% demand. Techniques offering reliable real-time monitoring with adequate specificity and accuracy occupy the 50% demand level. Highly sophisticated techniques, such as HPLC, reside at the top of the pyramid with a demand level of 25%, a category to which both methods in this study belong. Although these methods require substantial chemical consumption and specialized handling, such limitations were significantly reduced in the AI-optimized approach. Despite their lower demand weighting, both methods remain highly efficient, robust, and suitable for quality control and biological analysis, rendering them appropriate choices for these applications. While chemometric techniques and sensor-based approaches may exhibit higher demand weighting, their sensitivity to instrumental parameter variations may lead to erroneous outcomes, limiting their reliability for routine analysis.

Method quality, referred to as “whiteness,” was assessed using the RGB12 algorithm, which aims to identify analytical techniques that achieve high accuracy while remaining environmentally responsible and cost-effective. The quality scores obtained for the conventional and AI-optimized methods were approximately 81% and 88%, respectively. The overall NQS index, calculated as the average of sustainability, need, and quality scores, yielded values of 45% for the conventional method and 55% for the AI-optimized method. This represents a 10% improvement in favor of the AI-assisted approach, reflecting its superior optimization efficiency and overall performance, Table 5.

Collectively, these findings underscore the enhanced sustainability, quality, and analytical relevance of the AI-optimized method, as well as its stronger alignment with the SDGs. This work illustrates how analytical innovation can be effectively integrated with sustainability principles and emphasizes the importance of incorporating AI-driven strategies into modern analytical chemistry. Moreover, it highlights the broader value of international collaboration and methodological optimization in advancing sustainable scientific practices toward the 2030 global agenda.

Therefore, sustainability in the present work is not inferred solely from reduced solvent consumption, but is quantitatively assessed through integrated metrics that encompass greenness, analytical quality, operational efficiency, and societal impact.

**Table 4:** The sustainable aspects of proposed HPLC method, and its contribution to the UN’s sustainable development goals.

SDG	Remarks
GOAL 3: Good Health and Well-being	A robust system for comprehensive multi-drug analysis was developed to enhance health monitoring, reduce analytical errors, enable assays in biological fluids, and ensure improved quality control.
GOAL 4: Quality Education	This initiative promotes advancement in education and research while broadening the scope of analytical chemistry and sustainability.
GOAL 5: Gender Equality	This project demonstrates inclusive and equitable involvement of researchers across all genders.
GOAL 8: Decent Work and Economic Growth	This strategy offers a practical means of lowering economic costs by enabling more efficient and faster analytical processes.
GOAL 9: Industry, Innovation and Infrastructure	By providing a versatile technique applicable to the analysis of various dosage forms, this approach reinforces collaboration between academic research and the pharmaceutical industry while substantially conserving time and resources.
GOAL 11: Sustainable Cities and Communities	This approach incorporates time- and cost-efficient processes with reduced organic solvent consumption, achieving dual benefits of enhanced laboratory performance and minimized environmental impact.
GOAL 12: Responsible Consumption and Production	This approach provides a practical and cost-effective solution by reducing waste and eliminating hazardous solvents, thereby saving time, effort, and financial resources.
GOAL 13: Climate Action	This technique emphasizes environmental safety by avoiding hazardous solvents and minimizing waste, while also conserving time, labor, and costs.
GOAL 17: Partnerships to achieve the Goal	A unified, multi-purpose method for analyzing various drug formulations streamlines the analytical process, reduces time and costs, and fosters stronger collaboration between academic institutions, pharmaceutical companies, and biological entities.

**Table 5:** NQS index data output.

	Conventional-optimized method	AI-optimized method
Need	25	25
Quality	81	88
Sustainability	29	**53**
NQS Index	45	**55**

## Conclusion

The present work introduces a novel HPLC–DAD method for the simultaneous quantification of ranolazine, amlodipine, and diltiazem—three drugs frequently co-administered in chronic angina and hypertension therapy. This simultaneous assay fills a clear methodological gap and offers a valuable analytical tool for quality control laboratories, particularly if binary or tertiary dosage forms are introduced to the market, in addition to supporting future pharmacokinetic and clinical studies. Through the integration of AI-assisted optimization and multi-trait sustainability evaluation, the method demonstrates superior analytical performance, environmental compatibility, and practical applicability in both bulk, pharmaceutical dosage forms, and biological matrices. The proposed assay fulfills ICH validation standards with high sensitivity, accuracy, and reproducibility, while reducing solvent waste and energy consumption. Its outstanding eco-efficiency, confirmed by the MA assessment tool, positions it as a model for intelligent, sustainable, and future-ready analytical development. This approach not only meets pharmaceutical quality demands but also aligns with global sustainability goals, providing a blueprint for next-generation chromatographic method design. The AI-optimized method outperformed conventional chromatographic technique in both quality and sustainability scores using the NQS tool. Ultimately, these findings pave the way for further research by showcasing the potential of advanced AI tools to enhance the throughput and cost of pharmaceutical analysis.

## Supplementary Information

Below is the link to the electronic supplementary material.

Supplementary Material 1

Supplementary Material 2

Supplementary Material 3

Supplementary Material 4

Supplementary Material 5

Supplementary Material 6

Supplementary Material 7

## Data Availability

All data generated or analyzed during this study are included in this published article.

## Abbreviations

CVDs: Cardiovascular diseases
RNZ: Ranolazine
AMD: Amlodipine
DTZ: Diltiazem
MA tool: Multi-color assessment tool
SDGs: Sustainable development goals
NQS: Need, quality, and sustainability
MeOH: Methanol
ACN: Acetonitrile
IACUC: Institutional animal care and use committee
LOD: Limit of detection
LOQ: Limit of quantitation
Cmax: peak plasma concentrations
WAC: White analytical chemistry
GEMAM: Green evaluation metric for analytical methods
BAGI: Blueness assessment graphical index
RAPI: Redness analytical performance index
VIGI: Violet innovation grade index
AQbD: Analytical quality by design
