# Supplementary material for: Comparative AI-optimized HPLC–DAD strategy for the simultaneous determination of ranolazine, amlodipine, and diltiazem with pharmacotherapeutic relevance and multi-trait sustainability assessment
Source: Sci Rep. 2026 Apr 25;16:13407. doi: 10.1038/s41598-026-48679-w (PMC13110367; doi:10.1038/s41598-026-48679-w)
Supplement: Supplementary file 3 — Supplementary Material 3 [file 41598_2026_48679_MOESM3_ESM.pptx]

## Slide 1
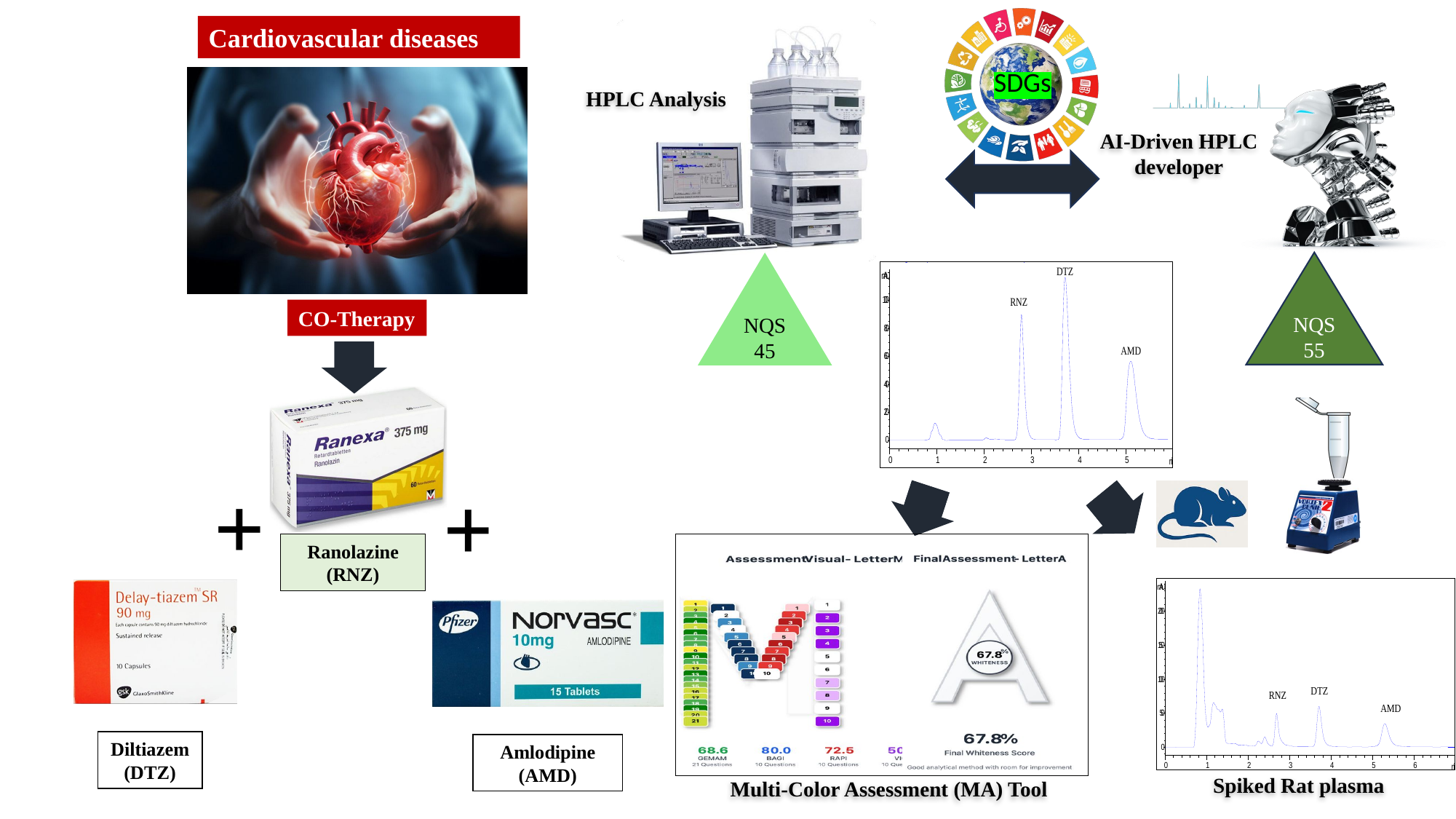

Cardiovascular diseases
HPLC Analysis
SDGs
AI-Driven HPLC developer
NQS
55
NQS
45
CO-Therapy
Ranolazine (RNZ)
Diltiazem (DTZ)
Amlodipine (AMD)
Spiked Rat plasma
Multi-Color Assessment (MA) Tool
