# Supplementary material for: Comparative AI-optimized HPLC–DAD strategy for the simultaneous determination of ranolazine, amlodipine, and diltiazem with pharmacotherapeutic relevance and multi-trait sustainability assessment
Source: Sci Rep. 2026 Apr 25;16:13407. doi: 10.1038/s41598-026-48679-w (PMC13110367; doi:10.1038/s41598-026-48679-w)
Supplement: Supplementary file 6 — Supplementary Material 6 [file 41598_2026_48679_MOESM6_ESM.pdf]

MA Assessment Tool Report

Multi-Color Assessment Index

M + A Visual Identity System

Assessment Results Summary

Final Whiteness Score: 64.8%

GEMAM (Green Experimental Matrix for Analytical Methods): 66.7%

BAGI (Blue Applicability Grade Index): 72.5%

RAPI (Red Analytical Performance Index): 70.0%

VIGI (Violet Innovation Grade Index): 50.0%

Performance Interpretation

Good - Strong performance with minor areas for improvement

Detailed Question Analysis

GEMAM - Green Experimental Matrix for Analytical Methods

Score: 66.7%

Q1: Where is the sample prepared?
Answer: Ex situ (Score: 1.19)

Q2: Is the sample damaged during preparation?
Answer: Yes (Score: 2.38)

Q3: What extraction mode is used?
Answer: Ultra-microextraction (Score: 4.76)

Q4: What is the sample size?
Answer: <10 g/mL (Score: 4.76)

Q5: How is the sample stored?
Answer: Frozen (Score: 1.9)

Q6: Is derivatization required?
Answer: No derivatization needed (Score: 4.76)

Q7: What are the hazards of the reagents used?
Answer: Low hazard level (Score: 3.57)

Q8: How much reagent is used per analysis?
Answer: Minimal (<1 mL) (Score: 4.76)

Q9: How many analytes are detected in a single run?
Answer: 2-3 analytes (Score: 1.9)

Q10: What is the sample throughput per hour?
Answer: 6-10 samples/hour (Score: 3.57)

Q11: How many steps are involved in the method?
Answer: 4 steps (Score: 3.81)

Q12: What percentage of materials are sustainable?
Answer: 15-45% sustainable (Score: 1.19)

Q13: What is the cost per sample analysis?
Answer: 1-10 USD (Score: 3.57)

Q14: What is the energy consumption per analysis?
Answer: Low consumption (0.1-0.5 kWh) (Score: 3.57)

Q15: Is the instrument automated?
Answer: Semi-automated (Score: 3.14)

Q16: Is the instrument miniaturized?
Answer: No miniaturization (Score: 2.38)

Q17: How is the waste treated?
Answer: Untreated disposal (Score: 0)

Q18: What is the hazard level of waste produced?
Answer: Low hazard waste (Score: 3.57)

Q19: How much waste is produced per analysis?
Answer: Minimal (<10 mL) (Score: 4.76)

Q20: Are the procedures hermetically sealed?
Answer: Yes, fully sealed (Score: 4.76)

Q21: How noisy is the analytical process?
Answer: Moderate noise (60-80 dB) (Score: 2.38)

BAGI - Blue Applicability Grade Index

Score: 72.5%

Q22: What type of analysis does the method provide?
Answer: Quantitative only (Score: 7.5)

Q23: How many analytes can be simultaneously determined?
Answer: 2-5 analytes of same class (Score: 5)

Q24: What analytical technique and instrumentation is used?
Answer: Simple/common instruments (UV, HPLC-UV, GC-FID) (Score: 7.5)

Q25: How many samples can be simultaneously treated?
Answer: 1 sample only (Score: 2.5)

Q26: What type of sample preparation is required?
Answer: Simple/low-cost (protein precipitation) (Score: 7.5)

Q27: What is the total throughput in samples per hour?
Answer: 5-10 samples/hour (Score: 7.5)

Q28: What type of reagents and materials are used?
Answer: Common and commercially available (Score: 10)

Q29: Is preconcentration required for the analysis?
Answer: Required, achieved in one step (Score: 7.5)

Q30: What is the degree of automation?
Answer: Semi-automated (common systems) (Score: 7.5)

Q31: What amount of sample is required?
Answer: "d100 ¼L/mg (bio) or "d10 mL/g (food/env) (Score: 10)

RAPI - Red Analytical Performance Index

Score: 70.0%

Q32: What is the repeatability (RSD%) of the method?
Answer: <1.0% (Score: 7.5)

Q33: What is the intermediate precision of the method?
Answer: <1.0% (Score: 10)

Q34: What is the reproducibility of the method?
Answer: <1.5% (Score: 10)

Q35: What is the trueness (bias%) of the method?
Answer: <2% (Score: 7.5)

Q36: What is the recovery and matrix effect performance?
Answer: >98% recovery (Score: 7.5)

Q37: What is the limit of quantification relative to expected levels?
Answer: LOQ <3% of expected mean (Score: 7.5)

Q38: What is the working range (upper limit/LOQ ratio)?
Answer: >30×LOQ (Score: 7.5)

Q39: What is the linearity (R^2) of the calibration curve?
Answer: $R^2 > 0.97$ (Score: 7.5)

Q40: How many factors were tested for ruggedness/robustness?
Answer: "e2 factors tested (Score: 5)

Q41: How many interferents were tested for selectivity?
Answer: No interferents tested (Score: 0)

VIGI - Violet Innovation Grade Index

Score: 50.0%

Q42: Does the method use advanced sample preparation or instrumentation (e.g., SPME, LIS, DLLME, HRMS, MS/MS)?
Answer: No, conventional approach (Score: 0)

Q43: Does it incorporate innovative data processing tools (AI, ML, in silico, blockchain, bioinformatics) and/or apply structured AQbD tools such as Design of Experiments (DoE), multivariate optimization, and statistical risk analysis?
Answer: Yes, high innovation (Score: 10)

Q44: Does it consider White Analytical Chemistry principles or metrics (AGREE, BAGI, RAPI, ComplexGAPI, MoGAPI)?
Answer: Yes, high innovation (Score: 10)

Q45: Does it address guidance from relevant regulatory organizations or legal bodies?
Answer: Yes, high innovation (Score: 10)

Q46: Does it use innovative reagents (e.g., MOFs, ILs, DES, 3D-printing, carbon dots, MIPs)?
Answer: No, conventional reagents (Score: 0)

Q47: Does it integrate miniaturized devices (portable systems, microfluidics, lab-on-chip, smartphones)?
Answer: No, conventional devices (Score: 0)

Q48: Does it integrate automation (robotics, on-flow systems, automated prep or online injection)?
Answer: Yes, moderate innovation (Score: 5)

Q49: Can the method be used across different fields (e.g., pharma, food, environment)?
Answer: Yes, moderate interdisciplinarity (Score: 5)

Q50: Does it significantly improve LOD/LOQ values (nano or pico-level detection)?
Answer: No, conventional sensitivity (Score: 0)

Q51: Does it offer a new application, matrix, target analyte, or theoretical idea (e.g., hot topics)?
Answer: Yes, high novelty (Score: 10)
