# Supplementary material for: Comparative AI-optimized HPLC–DAD strategy for the simultaneous determination of ranolazine, amlodipine, and diltiazem with pharmacotherapeutic relevance and multi-trait sustainability assessment
Source: Sci Rep. 2026 Apr 25;16:13407. doi: 10.1038/s41598-026-48679-w (PMC13110367; doi:10.1038/s41598-026-48679-w)
Supplement: Supplementary file 7 — Supplementary Material 7 [file 41598_2026_48679_MOESM7_ESM.docx]

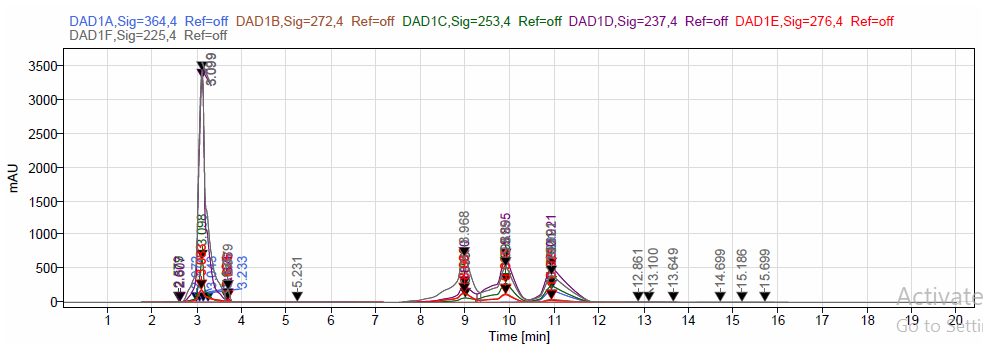


Fig. S1. Chromatogram showing late elution of the studied drugs using C8 column (9-11 min)

RNZ

DTZ

b)

a)

AMD

DTZ

AMD

RNZ

Fig. S2. Chromatogram of the ratio of the dosage form RNZ 40, DTZ 9, AMD 1 µg/mL at a) 240 nm and b) 270 nm

AMD

DTZ

RNZ

Fig. S3. chromatogram showing spiked plasma followed by acetonitrile protein precipitation

a)

b)c)

Fig. S4. Purity profiles and plots of RNZ (a), DTZ (b) and AMD (c)

Fig. S5. chromatogram showing spiked plasma at concentration 1 µg/mL of RNZ, DTZ and AMD

AMD

DTZ

RNZ

AMD

DTZ

RNZ

RNZ

Fig. S6. chromatogram showing spiked plasma at concentration 5 µg/mL of RNZ, DTZ and AMD

**Table S1: Evaluation of the robustness of the proposed HPLC method for the determination of RNZ, DTZ and AMD.**

| **Parameters** | **RNZ Mean % recovery ± SDᵃ** | **RSD%ᵇ** | **t_R_± SDᶜ** | **DTZ Mean % recovery ± SDᵃ** | **RSD%ᵇ** | **t_R_ ± SDᶜ** | **AMD Mean % recovery ± SDᵃ** | **RSD%ᵇ** | **t_R_ ± SDᶜ** |
| --- | --- | --- | --- | --- | --- | --- | --- | --- | --- |
| **Temperature (25 ± 2 ºC)** | 100.12 ± 0.45 | 0.45 | 2.79 ± 0.06 | 99.86 ± 0.38 | 0.38 | 3.72 ± 0.05 | 100.34 ± 0.41 | 0.41 | 5.15 ± 0.07 |
| **Wavelength of determination (selected λ ± 1 nm)** | 99.78 ± 0.29 | 0.29 | – | 100.21 ± 0.33 | 0.33 | – | 99.92 ± 0.27 | 0.27 | – |
| **pH of aqueous phase (5 ± 0.2 units)** | 100.43 ± 0.35 | 0.35 | 2.79 ± 0.05 | 99.91 ± 0.42 | 0.42 | 3.72 ± 0.06 | 100.15 ± 0.37 | 0.37 | 5.15 ± 0.06 |

ᵃ Mean percentage recovery of peak area of RNZ, DTZ, and AMD (10 μg/mL) under the studied conditions ± SD.
ᵇ Percentage relative standard deviation of % recovery for RNZ, DTZ, and AMD at the studied conditions.
ᶜ Mean retention time at the studied conditions ± SD.

**Table S2 Determination of RNZ, DTZ and AMD in pharmaceutical dosage form**

| Drug | Amount taken (µg/mL) | Amount found* (µg/mL) ± SD | % Recovery | %RSD |
| --- | --- | --- | --- | --- |
| RNZ | **40** | **39.42 ± 0.35** | **98.56** | **0.89** |
| DTZ | **9** | **9.11 ± 0.05** | **101.25** | **0.55** |
| AMD | **1** | **0.99 ± 0.02** | **99.32** | **1.63** |
| *The mean of five determinations | | | | |
